# Re-distribution of oxygen at the interface between γ-Al_2_O_3_ and TiN

**DOI:** 10.1038/s41598-017-04804-4

**Published:** 2017-07-03

**Authors:** E. O. Filatova, A. S. Konashuk, S. S. Sakhonenkov, A. A. Sokolov, V. V. Afanas’ev

**Affiliations:** 1 Institute of Physics, St-Petersburg State University, Ulyanovskaya Str. 1, Peterhof 198504, St. Petersburg, Russia; 2 Helmholtz-Zentrum Berlin für Materialien und Energie GmbH, Albert Einstein Str. 15, 12489 Berlin, Germany; 3 Department of Physics, University of Leuven, Celestijnenlaan 200D, 3001 Leuven, Belgium

## Abstract

Interface of TiN electrode with γ-Al_2_O_3_ layers was studied using near edge X-ray absorption fine structure, conventional X-ray photoelectron spectroscopy and photoelectron spectroscopy with high energies. Despite the atomic-layer deposited Al_2_O_3_ being converted into thermodynamically-stable polycrystalline cubic γ-phase by high-temperature (1000 or 1100 °C) anneal, our results reveal formation of a thin TiN_x_O_y_ (≈1-nm thick) interlayer at the interface between γ-Al_2_O_3_ film and TiN electrode due to oxygen scavenging from γ-Al_2_O_3_ film. Formation of the TiO_2_ was not observed at this interface. As environmental effect, a strong oxidation resulting in formation of a TiO_2_(1.4 nm)/TiN_x_O_y_(0.9 nm) overlayers on the top of the TiN electrode is traced. Development of O-deficiency of γ-Al_2_O_3_ is observed and related to the polarization anisotropy due to the preferential orientation of spin states involved in the X-ray absorption in the plane parallel to the surface. Investigation of the TiN electrode reveals the predominantly “stretched” octahedra in its structure with the preferential orientation relative the interface with γ-Al_2_O_3_. This anisotropy can be correlated with ≈200 meV electron barrier height increase at the O-deficient TiN/γ-Al_2_O_3_ interface as compared to the TiN/γ-Al_2_O_3_ barrier formed under abundant oxidant supply condition as revealed by internal photoemission of electrons from TiN into the oxide.

## Introduction

Since reduction of energy dissipated in semiconductor integrated circuits (ICs) requires lowering of the power supply voltage down to few hundreds of mV, it becomes critically important to control the internal electric fields in the multi-layer device structures. In particular, tight control of the built-in voltages arising from the effective work function (EWF) differences between electrodes represents fundamental challenge because it directly contributes to the electric field in the active device region (transistor channel, conducting filament in resistive memory cell, etc.). As a result, the EWF must not only be precisely tailored to achieve the desired performance but it should also remain stable during device operation to ensure long-term reliability. However, in reality, significant processing-induced EWF variations amounting to hundreds of meV have been reported over decades. The EWF variations have been associated with formation of dipoles in metal-insulator-semiconductor (MIS) structures, including dipoles at the high-κ oxide/SiO_2_ interface^1^, charged oxygen vacancies in the high-κ oxide^2^ as well as dipoles induced by post-metallization anneal^3–5^. Furthermore, in the case high-κ oxide insulators used in modern ICc such as HfO_2_, generation of oxygen imbalance at the interface by introducing metal scavenging layer is shown to result in nearly 1 eV metal/oxide barrier variations^6, 7^. The O loss from the thermodynamically-stable γ-Al_2_O_3_ phase has recently been reported to be induced by a simple metallization (TiN) process^8^. While the mentioned observations leave no doubt that oxygen re-distribution at the metal/oxide interface represents the major driving force of interface barrier and EWF variation, atomic picture of this crucial process remains incomplete: most of the attention has been devoted so far to the electron states inside the near-interface insulating layer leading to the break of electro-neutrality while the “fate” of oxygen leaving the insulating film as well as its influence on the EWF remains unknown.

The complexity of the problem is related to formation of interlayers (ILs) at the metal/insulator interfaces due to interdiffusion and chemical reactions during the synthesis of such systems. Analysis of different metal-oxide-semiconductor (MOS) gate stacks reveals the increasingly important role of such ILs, which may impact the functionality of the devices by, for example, affecting the EWF of electrodes^9–11^. According to ref. 12, an additional complication arises from the formation of a “polarization layer” associated with charges located in the insulator close to the metal surface. Furthermore, Mead and co-authors were the first who measured^13, 14^ the metal/SiO_2_ barrier energies and have proposed an alternative model of the polarization layer, which invokes penetration of the field into the metal.

To progress towards better understanding the physics and chemistry of γ-Al_2_O_3_/TiN interface barrier formation, in the present work we addressed the physical mechanism behind the metal/insulator barrier variations for the case of TiN/γ-Al_2_O_3_ interfaces in practically relevant TiN/γ-Al_2_O_3_/TiN and TiN/γ-Al_2_O_3_/Si stacks used in charge trapping (flash) memory cells^15, 16^. Nowadays the (poly) crystalline γ-Al_2_O_3_ layers offer advantageous alternative to the amorphous (a-) alumina films widely applied in a variety of electron devices. For example, the increase of the conduction band (CB) offset at interfaces with semiconductors upon alumina crystallization^17, 18^ allows for better gate insulation of wide-bandgap channel materials such as GaN^19^ and SiC^20, 21^. In particular, polycrystalline γ-Al_2_O_3_ films obtained by annealing-induced crystallization of the atomic-layer-deposited (ALD) alumina^17, 22^ are seen as superior inter-gate insulator in charge trapping memory cells^23^ eventually allowing for 3-dimensional integration, improving the cell performance^23^, as well as promising a high-temperature operation^24^. However, retention properties of the Al_2_O_3_-insulated memory cells appear to critically depend on the oxygen deficiency developed during post-deposition anneal mandating application of an oxygen-containing ambient^25^. As we have established earlier^8^, γ-Al_2_O_3_ layers obtained by high-temperature crystallization anneal may develop oxygen deficiency upon the later processing steps carried out at significantly lower temperature. The near-edge X-ray absorption fine structure (NEXAFS) experiments reveal that even in the initially stoichiometric γ-Al_2_O_3_ the room-temperature plasma-enhanced deposition of metal electrodes (TiN or TaN) leads to formation of O deficient region in the oxide^8^ and gap states, which may cause electron leakage current.

Analysis of the atomic and electronic structures reveals the formation of thin TiN_x_O_y_ (≈1 nm thick) layer at interface between γ-Al_2_O_3_ film and the top TiN electrode as a result of oxygen scavenging from the γ-Al_2_O_3_ while no TiO_2_ phase was found. This chemical modification of the near-interfacial metal layer can now clearly be separated from formation of TiO_2_(1.4 nm)/TiN_x_O_y_(0.9 nm) layers on the top electrode surface in the TiN/γ-Al_2_O_3_/TiN stack which represents an artifact of ambient exposure. The oxygen uptake in the near-interface TiN layer results in different atomic structures of top and bottom TiN/γ-Al_2_O_3_ interfaces in seemingly symmetric TiN/γ-Al_2_O_3_/TiN capacitor stack. As a result, the interface barrier height at the top (Ο-deficient) interface appears by ≈200 meV higher than at the bottom interface formed under excess of oxidant during ALD of alumina. This barrier height difference corresponds to EWF difference of TiN at two chemically different interfaces and will be translated into non-zero built-in voltage across seemingly “symmetric” TiN/Al_2_O_3_/TiN stack.

## Results

### High kinetic photoemission studies (HAXPES)

The sample preparation procedures and the structure geometry are summarized in the first paragraph of the “Methods” section. A key factor in any photoemission experiment is the sample probing depth. It is traditionally related to the inelastic mean free path (IMFP, λ_i_)^26^, which is defined as the average distance that an electron with a given kinetic energy travels between successive inelastic collisions. The main bonus of HAXPES consists in a large value of λ_i_ providing in depth analysis of nanosystems, which was realized in the current studies. The photoelectron spectra were measured at fixed excitation energy by changing the electron emission angle. The choice of the excitation energy was dictated by the main objective of this study, i.e., the in-depth probing of the TiN/γ-Al_2_O_3_ stack including the interface between the dielectric layer and the electrode through the whole electrode thickness (10 nm). For example, the IMFP calculated by means of TPP-2M formula^27^ for TiN amounts to 4 nm for Ti2p photoelectrons excited by 3010 eV photons. The maximum probing depth is defined as (3 λ_i_ cosθ), which corresponds to ≈95% of the total signal intensity to be formed within the layer of this thickness. The analysis of Al 2s line measured at different angles using excitation energy of 3010 eV reveals a distinct signal from this line at the emission angles 5° and 20° (not shown). Therefore, the selected excitation energy allowed us to study the sample without removing the 10-nm thick TiN electrode by changing the electron emission angle.

**Figure 1:**
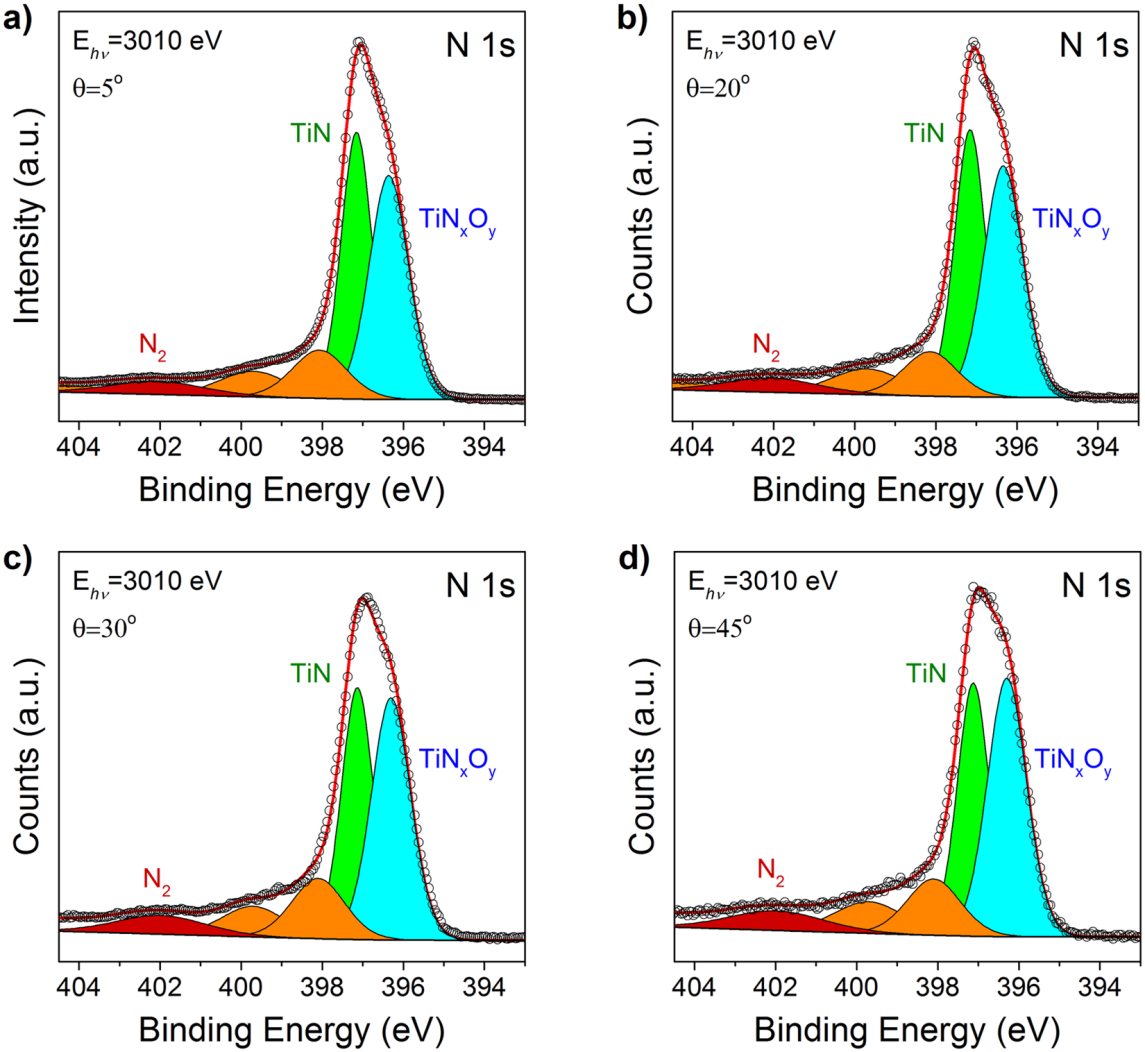
Experimental and fitted N1s photoelectron spectra from TiN/Al_2_O_3_/Si stack measured at an excitation photon energy of 3010 eV and different electron emission angles: (**a**) 5°; (**b**) 20°; (**c**) 30° and (**d**) 45°.

**Figure 2:**
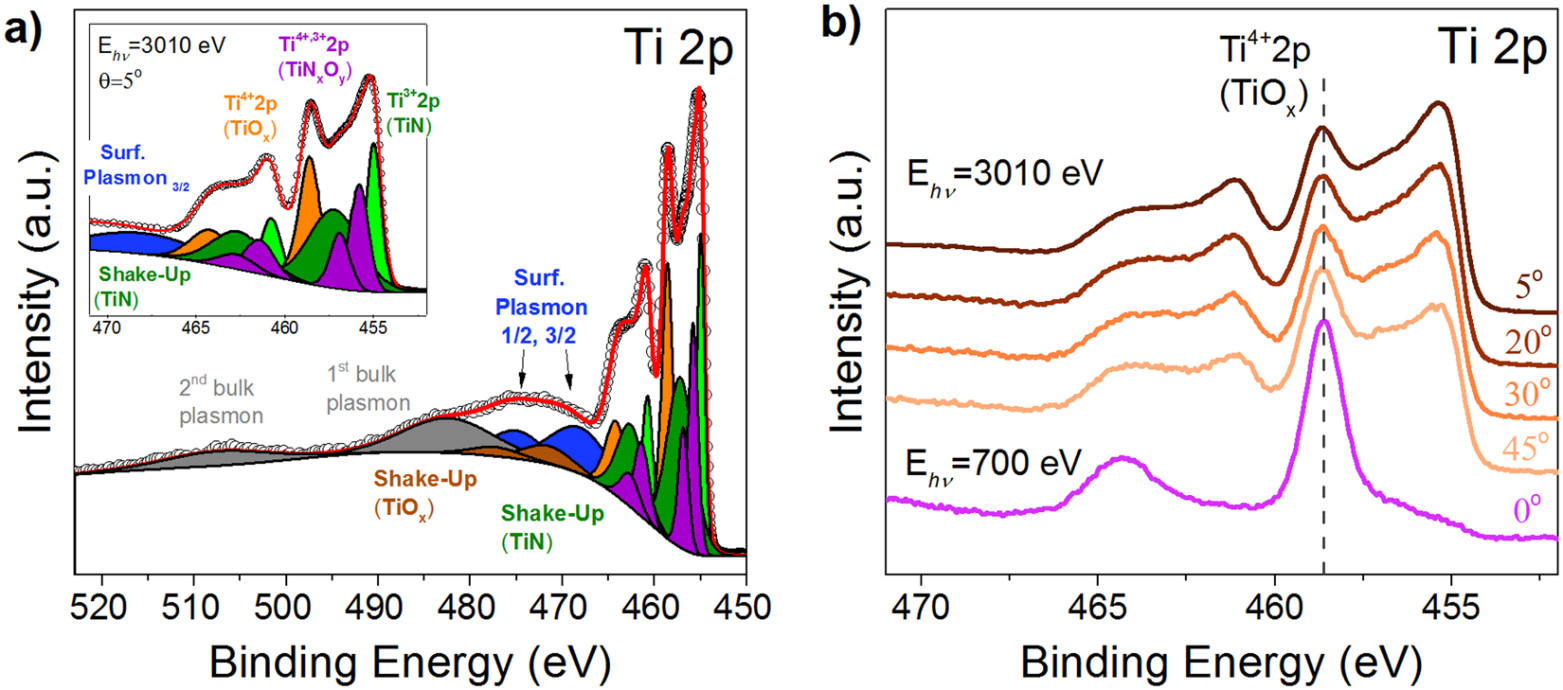
Experimental Ti2p photoelectron spectra collected from TiN/γ-Al_2_O_3_/Si stack. Panel (a) shows the experimental and fitted spectrum measured at the excitation energy of 3010 eV and electron emission angle of 5°. The left hand inset is zooming in the main part of the spectrum. Panel (b) shows the spectra measured at excitation energy of 3010 eV and electron emission angles of 5°, 20°, 30° and 45° and the spectrum measured at the excitation energy of 700 eV and normal emission. All spectra in panel (b) are normalized to the background providing the same intensity and are shifted along the ordinate for clarity.

Experimental N1s and Ti2p photoelectron spectra collected from a TiN/Al_2_O_3_/Si stack at the indicated excitation energy of 3010 eV and different electron emission angles are shown in Figs 1 and 2. All the spectra were normalized to the incident photon flux, the illuminated sample area, and transmission function of the electron energy analyzer^28^. The fitting was realized using CASA XPS to resolve different components of the spectra.

Figure 1 compares the N1s HAXPES spectra measured at different electron emission angles. The universal cross section Tougaard background was used with limits set from 390 eV to 435 eV. Analysis of these spectra reveals two major peaks in the main band: The peak at 397.1 eV is assigned to TiN phase while the additional peak observed at the lower binding energy (396.4 eV) indicates the TiN_x_O_y_ phase formation^29–31^. The presence of the peak at 396.4 eV suggests significant oxidation of the electrode material. One can see that the intensity ratio of the peaks stemming from TiN and TiN_x_O_y_ phases depends on the electron emission angle. The relative TiN peak intensity with respect to the peak from TiN_x_O_y_ phase becomes larger with increasing the probing depth (decrease of the electron emission angle) and can be ascribed to the formation of TiN_x_O_y_ overlayer on the top of TiN electrode as a result of oxidation in air^29, 31^. Other higher energy peaks are related to different molecular nitrogen species, which are inherent features characteristic of TiN and other transition-metal nitrides^29, 31–33^. While these peaks are always present at approximately the same energy positions as reported in literature^29, 31, 32, 34^, the exact assignment of the peaks shown in orange (398 eV and 399.8 eV) remains ambiguous. Additional dedicated experiments will probably be needed to clarify their nature. By contrast, the peak shown in red at 402 eV can be unequivocally related to molecular nitrogen N_2_^35^ as will be discussed later in this paper.

Figure 2 compares the Ti2p HAXPES spectra measured at excitation energy of 3010 eV and different electron emission angles. Only the decomposition of the Ti2p spectrum measured at electron emission angle of 5° is shown as an example. The decomposition of the Ti2p spectra was performed using three parameter Tougaard background^36^ with parameters B and C fixed at 681 eV^2^ and 355 eV^2^ and parameter D adjusted to be 1800 eV^2^ to meet the background at 524 eV after all plasmon loss features. A standard Gauss–Lorentzian product line shape GL(30) was used to fit the spectra. Two groups of peaks were introduced to perform the decomposition procedure. The first group consists of four spin-doublet pairs of photoelectron lines (Ti2p_3/2_ components in the 455–459 eV range and Ti2p_1/2_ in the 460–466 eV range) and two pairs of shake-up satellites (457.4 eV–TiN shake-up^37, 38^ and 472.1 eV–TiO_2_ shake-up^39^). The second group is formed by the rather broad plasmon loss features introduced according to ref. 37. This group includes contributions of two surface plasmons excited by Ti 2p_3/2_ and Ti 2p_1/2_ photoelectrons, first and second order bulk plasmons.

Let us consider the first group of peaks. The main peak centered at 455.1 eV is assigned to TiN phase^30, 35, 37^. It should be noted that the energy separation between Ti2p_3/2_ and N1s photoelectron lines ΔE(Ti2p_3/2_, N1s) represents specific characteristic of TiN and reflects its stoichiometry^38^. The energy splitting ΔE(Ti2p_3/2_, N1s) in the measured spectra reaches 58.0 eV that corresponds to nearly stoichiometric TiN. This conclusion is additionally supported by observation of a shake-up satellite in Ti2p HAXPES spectra. It has been shown in ref. 38. that a shake-up satellite in Ti2p XPS is a characteristic of Ti2p spectrum of nearly stoichiometric TiN_x_ with x > 0.8. Therefore, in our decomposition the TiN peaks are followed by the shake-up satellite structure.

Next, let us consider in more detail the decomposition of the Ti2p spectrum. The shake-up energy loss was fixed at 2.2 eV for Ti2p_3/2_ and 1.8 eV for Ti2p_1/2_ according to results of ref. 37. Since the oxynitride peaks occur in the same binding energy range as the shake-up satellite^29, 31^ the area ratio between the shake-up satellite and the TiN peak was kept constant and equal to 1.8, as expected for nearly stoichiometric TiN_x_ (x > 0.8)^37^. The oxynitride peaks at 457.3 eV and 456.2 eV were introduced according to ref. 34. and correspond to Ti^4+^N_x_O_y_ and Ti^3+^N_x_O_y_ phases, respectively. The remaining photoelectron peak at 458.7 eV is attributed to Ti^4+^ chemical state in TiO_x_ phase^40, 41^. The lowest oxidation states of titanium in TiO_x_ phase were not introduced separately since their energy positions coincide with those of Ti oxynitride peaks^40, 41^. In the framework of this decomposition, the peaks originating from the Ti oxynitride provide information about all the intermediate chemical states of titanium atoms: both oxynitride states and lower oxidation states in TiO_x_ phase. The spin-orbit splitting for Ti2p line in TiO_x_ and for TiN and oxynitride peaks was fixed at 5.7 eV^41^ and 5.8 eV^37^, respectively. The obtained intensity ratios of the Ti2p_1/2_ and Ti2p_3/2_ doublets were 0.50 ± 0.04.

Using this decomposition analysis of Ti2p spectra shown in Fig. 2, a complex multiphase composition of the 10 nm TiN electrode related to its oxidation can be clarified. While the oxidation of TiN in air is well known^29, 31^ to result in few nm thick oxide and oxynitride layers, a possible oxygen scavenging from underlying oxide layer (γ-Al_2_O_3_) was first revealed in our previous work^8^. To evaluate the actual depth distribution of chemical states of titanium atoms and to determine the thicknesses of individual layers, which constitute the “real” TiN electrode, we used the approach described in details in our previous reports^40, 42^. Within this approach, the experimental peak area dependencies on the electron emission angle (at fixed excitation energy) are modeled by theoretical curves constructed based on the following recurrent formula for the intensity of an HAXPES peak from an *n* ^th^ layer in a multi-layer stack:

1 $${F}_{n}(\theta )={A}_{\exp }(\theta ){\sigma }_{n}{c}_{n}{\lambda }_{n}{\gamma }_{n}(1-{e}^{\frac{-{d}_{n}}{{\lambda }_{n}\cos (\theta )}})\prod _{i=1}^{n-1}{e}^{\frac{-{d}_{i}}{{\lambda }_{i}\cos (\theta )}}.$$

here, *θ* is the electron emission angle with respect to the sample normal, *n* numbers the structurally different layers (*n* = 1 corresponds to the upper layer), σ_*n*_ is the photoexcitation cross-section, λ_*n*_ is the inelastic mean free path calculated by means of TPP-2M formula^27^, γ_*n*_ is the orbital angular symmetry factor, *c*_*n*_ is the atomic concentration, and *d*_*n*_ is the thickness of the *n* ^th^ layer. The constant *c*(*z*) has the same value for each layer. *A*_exp_(θ) = *TA* cosθ, *T* is a constant accounting for the geometry of the experimental setup, and *A*_*exp*_ is the analyzed area of a sample. The values of the cross-sections and the asymmetry factors were taken from ref. 43.

**Figure 3:**
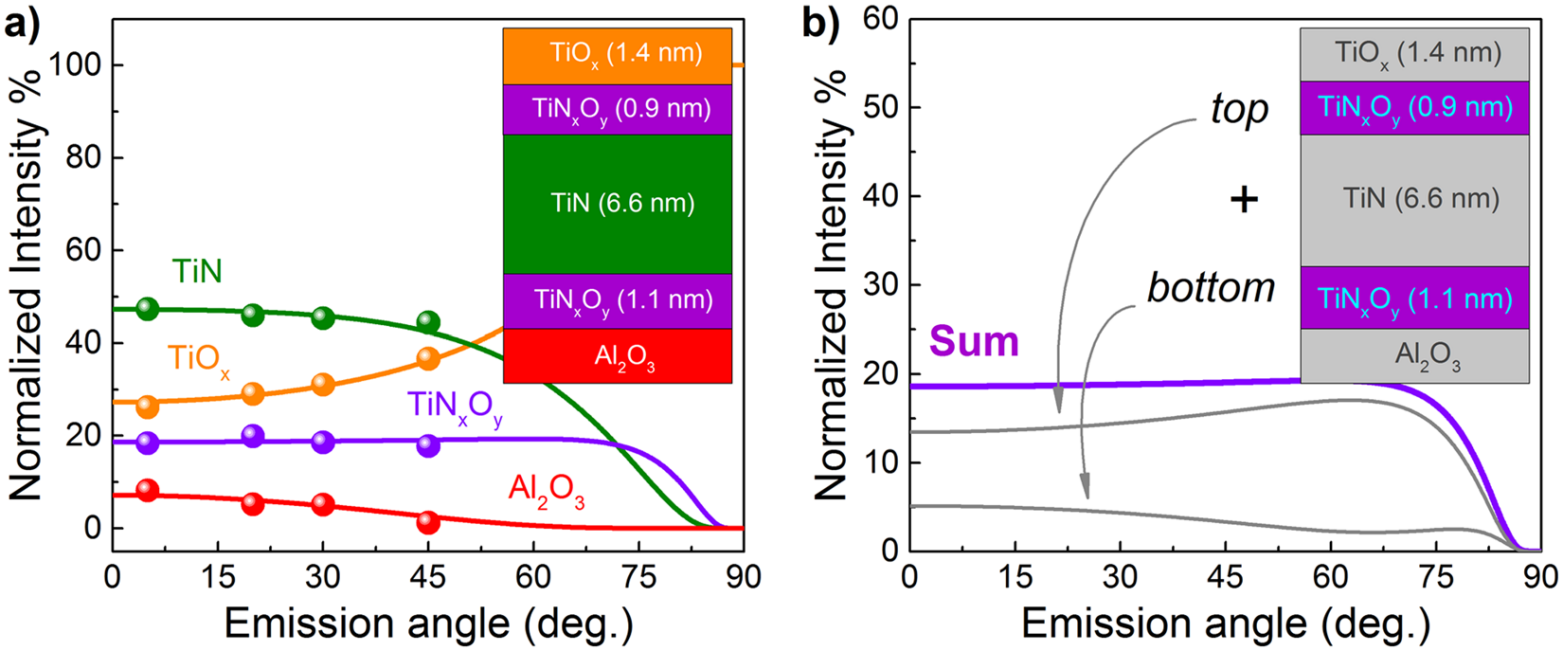
(**a**) Experimental (dots) and calculated (lines) values of HAXPES peak intensities for different electron emission angles using the five-layer model for TiN/Al_2_O_3_/Si sample (3010 eV excitation). For clarity, all the intensities are normalized using the constants σ, *c*, λ, γ and *A*exp(θ). Panel (b) illustrates the simulated angular behavior of peak intensities associated with electron emission from the top and bottom TiN_x_O_y_ layers and their resulting sum.

As follows from the Ti2p spectrum decomposition, the TiN electrode contains three chemically different phases and can be represented as a five-layered system where the very bottom layer corresponds to the underlying γ-Al_2_O_3_ film with the thickness exceeding the probing depth. Importantly, it appears to be impossible to fit the experimental results if assuming that TiN_x_O_y_ layer is present only on top of TiN without introducing additional TiN_x_O_y_ layer between TiN and γ-Al_2_O_3_. One can see from Fig. 3b that the simulated angular curve for the top TiN_x_O_y_ layer has the shape, which in principle cannot fit the experimental dependency shown in Fig. 3a since they exhibit opposite trends with increasing electron emission angle. Only a sum curve combining contributions of both top and bottom TiN_x_O_y_ layers fits the experimental angular profiles resulting in the layer structure of the “real” TiN electrode illustrated by the inset in Fig. 3a.

**Figure 4:**
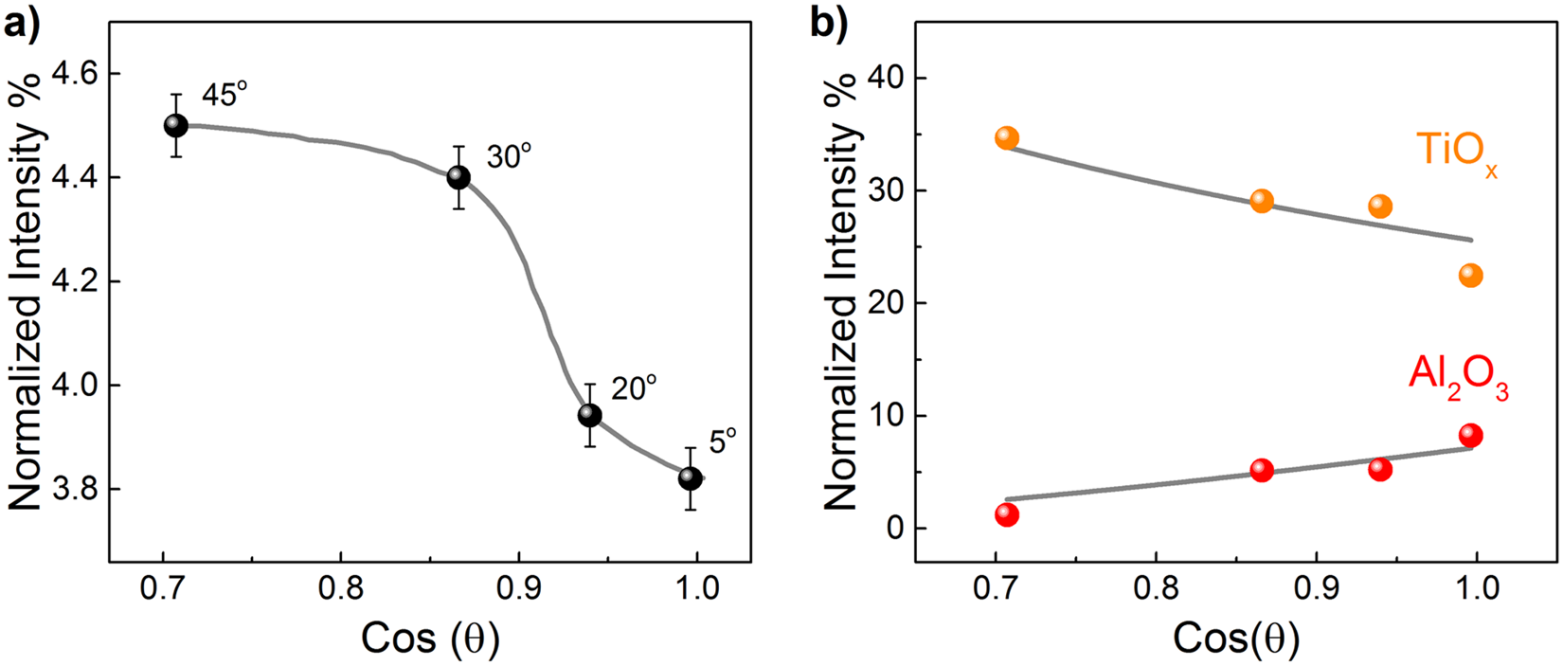
Relative fraction of N_2_ peak (centered at 402 eV) in the whole intensity of N1s line (**a**) and Ti^4+^ (TiO_x_) and Al2s (Al_2_O_3_) peaks (**b**) measured using 3010 eV excitation photons and different electron emission angles. The typical error margin was about 0.05%.

The above inference about TiN oxidation at the bottom TiN/γ-Al_2_O_3_ interface is independently supported by the analysis of angular dependence of the N1s photoemission spectra shown in Fig. 1. The observed N1s peak with binding energy of 402 eV is known to be related to formation of N_2_ molecules as a result of TiN oxidation^35^. Since N_2_ is expected to reside inside the oxidized TiN layer(s), we analyzed the intensity of the N_2_ emission peak as a function of electron emission angle. The relative intensity of this peak normalized to the total N1s line intensity is shown in Fig. 4a as a function of cosine of the electron emission angle. This dependence suggests the presence of an inflection point which may occur only if there are more than one N_2_-containing layer in the oxidized TiN electrode. Therefore, it is likely that formation of N_2_-containing oxidized regions occurs both at the top surface of TiN and at the TiN/γ-Al_2_O_3_ interface. For the sake of comparison, similar emission angular dependences corresponding to Ti^4+^ states in TiO_x_ and to Al2s states in γ-Al_2_O_3_ are shown in Fig. 4b. These dependences exhibit no sign of an inflection which agrees with the presence of TiO_x_ and Al_2_O_3_ as single layers in the studied sample.

Indeed, the surface nature of TiO_x_ layer is confirmed by comparison of the experimental Ti2p photoelectron spectrum from the TiN/Al_2_O_3_/Si stack measured at the excitation energy of 3010 eV and different electron emission angles to the spectrum taken at the excitation energy of 700 eV and normal to the surface direction of emission shown in Fig. 2b. Taking into account that the lower excitation energy corresponds to the smaller probing depth, one can conclude from the intensities of the Ti^4+^2p_3/2_–Ti^4+^2p_1/2_ peaks that TiO_x_ layer has been formed at the surface of the metallic TiN electrode due to oxidation in air.

All in one, the results discussed in this section leave no doubt about formation of thin TiN_x_O_y_ layer at the interface between γ-Al_2_O_3_ film and TiN electrode as a result of oxygen scavenging from the γ-Al_2_O_3_ film while the TiO_x_ phase is not observed at the interface.

### Al L_2,3_- and OK-absorption spectra of the γ-Al_2_O_3_ film

**Figure 5:**
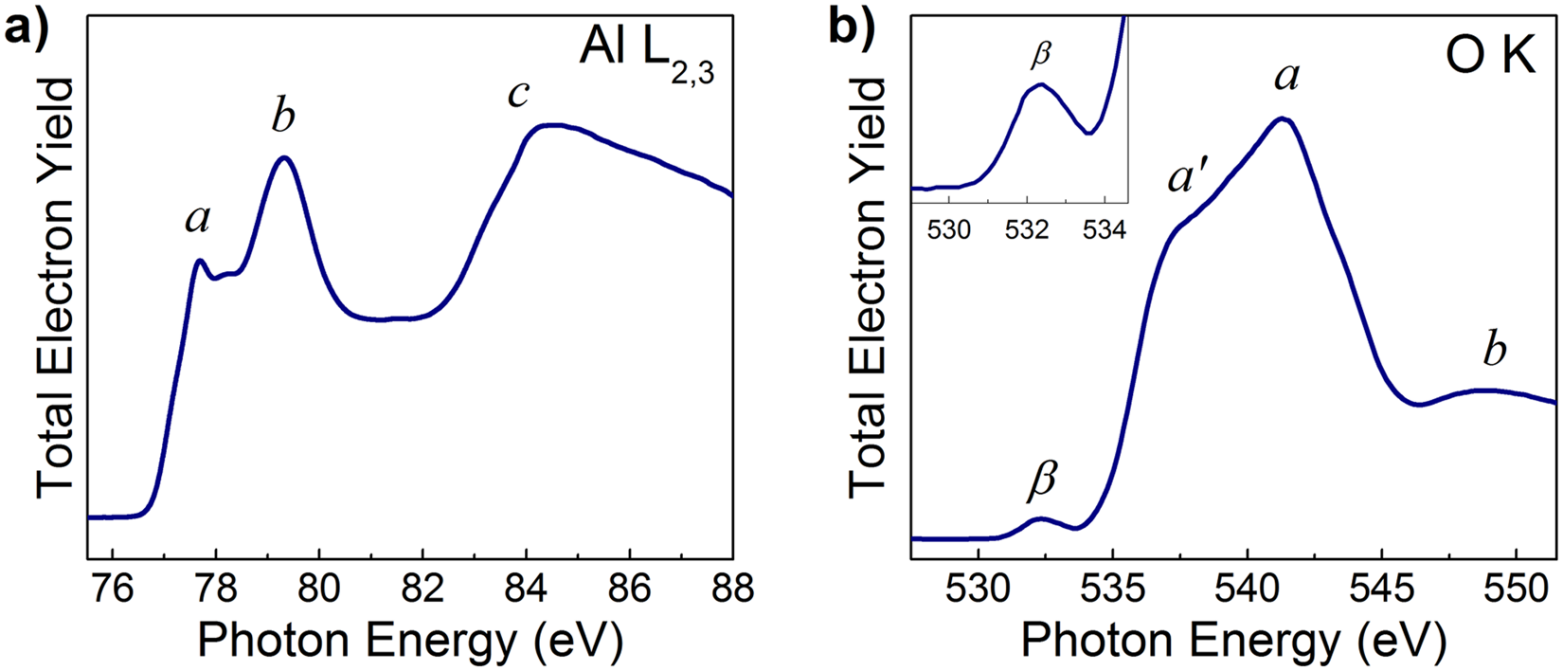
(**a**) Al L_2,3_- and (**b**) O K-absorption spectra of the γ-Al_2_O_3_ films measured outside the TiN electrode (after contact with electrode–see Methods section, end of the first paragraph) by monitoring the total electron yield when the drain current from the sample is measured.

To reveal the nature of the driving force behind formation of thin TiN_x_O_y_ layer at the interface between γ-Al_2_O_3_ film and TiN electrode we analyzed the interface from both sides, namely from the side of the oxide film and from the TiN electrode side. The Al L_2,3_- and O K-absorption spectra of the exposed γ-Al_2_O_3_ film measured outside the TiN electrode (see Methods section) and thereby providing the information about the structure of the γ-Al_2_O_3_ film after contact with electrode are shown in Fig. 5. The spectra were measured by monitoring the total electron yield by measuring the drain current from the sample. The measured absorption spectra were normalized to the continuum jump after subtraction of linear background extrapolated from the energy region below the absorption onset.

The fine structure of the Al L_2,3_- absorption spectrum of γ-Al_2_O_3_ (the main features are labeled ***a***−***c***, Fig. 5a) is determined by electron transitions from 2p states of Al to the unoccupied molecular orbitals (MOs) of the CB. Notice that each alumina phase exhibits a specific energy splitting ΔE between the features ***a*** and ***b***, reflecting the differences in effective charge on the Al atom. In its turn, the ratio between the intensities of ***a*** and ***b*** peaks corresponds to the relative abundance of the tetrahedrally and octahedrally coordinated Al atoms^44–46^. Analysis of the Al L_2,3_-absorption spectrum shown in Fig. 5a reveals the energy splitting ΔE_***a***−***b***_ = 1.6 eV and the intensity ratio I_***a***_/I_***b***_ = 0.72 (within the accuracy limit of ~0.01 as determined by the uncertainties of inferring the intensities and positions of the peaks ***a*** and ***b***), which corresponds to the γ-Al_2_O_3_ phase^46, 47^.

As follows from the calculation of the partial densities of states (PDOS)^48, 49^ the O K-absorption spectrum of alumina originates from electron transitions to the unoccupied states in the conduction band derived from the 2p states of O atoms mixed with Al e (t_2g_) and Al t_2_ (e_g_) states (main band ***a***′-***a***) and with Al 3s-, Al 3p-states (feature ***b***). Analysis of the O K-absorption spectrum reveals the latter to be in good agreement with the known spectrum of γ-Al_2_O_3_^19, 44, 46^. Of particular interest is the pre-edge region of the O K-absorption spectrum where additional feature ***β*** can be seen (Fig. 5b). As follows from refs 8, 50, 51. this feature ***β*** is associated with oxygen deficiency of the γ-Al_2_O_3_ layer, promoted by oxygen scavenging by active metal such as Ti during electrode sputtering. To exclude the possible effect of plasma damage of γ-Al_2_O_3_ layer during plasma etching of TiN electrode, wet etching of TiN in H_2_O_2_ was applied to the identical TiN/γ-Al_2_O_3_/Si sample, which is not expected to cause any oxygen loss. The O K-absorption spectrum measured in the exposed area of γ-Al_2_O_3_ film reveals the appearance of feature ***β*** in the spectrum even after H_2_O_2_ etching. Because this exposed area of γ-Al_2_O_3_ film obviously was not subjected to any plasma treatment, the feature ***β*** can now be associated with oxygen deficiency of the γ-Al_2_O_3_ sub-surface layer due to oxygen scavenging by TiN electrode.

**Figure 6:**
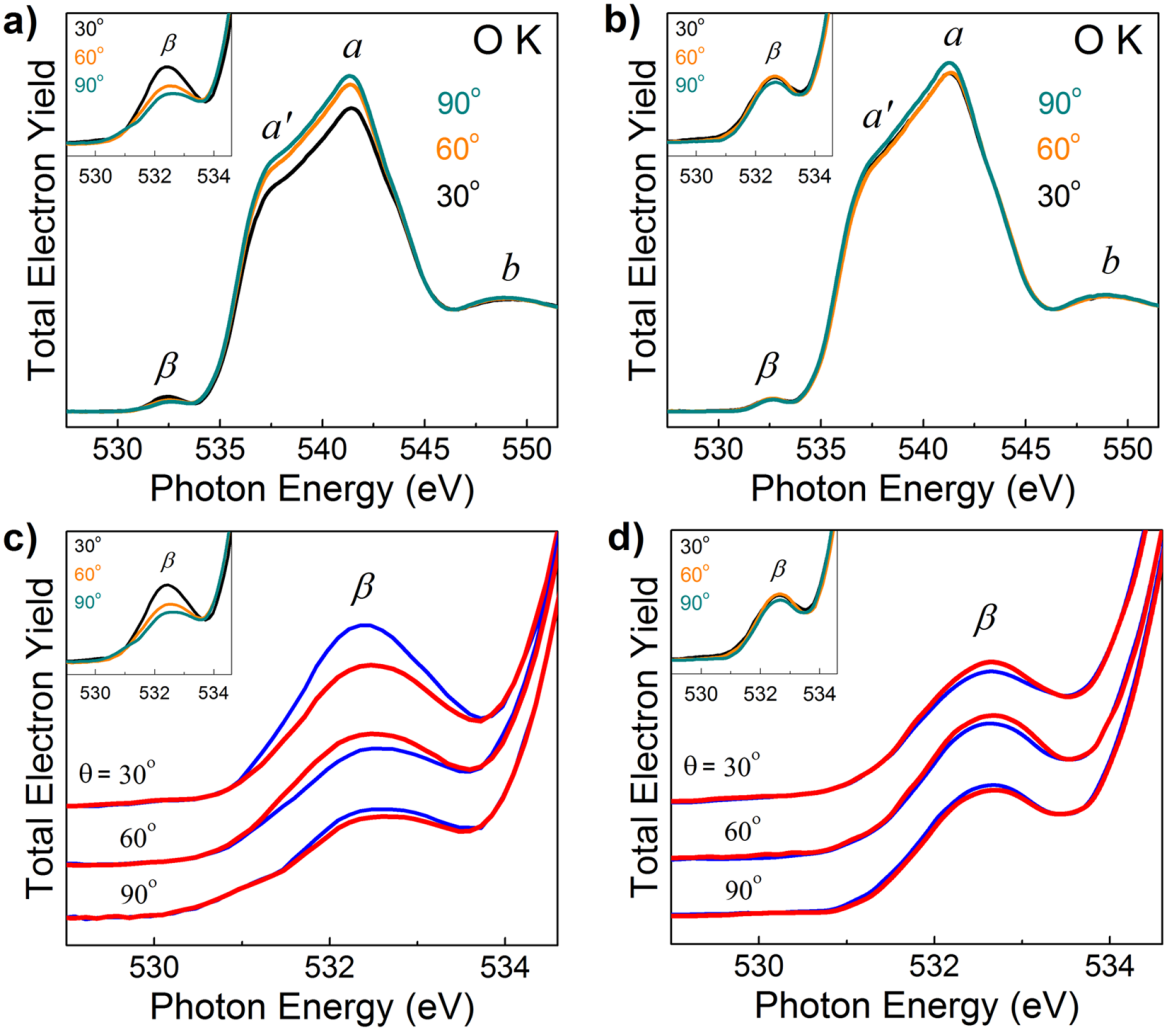
O K-absorption spectra of TiN/γ-Al_2_O_3_/Si sample measured at three different grazing incidence angles (30°, 60° and 90°) and two azimuth positions of the sample (φ = 0° and φ = 90°) using left (blue curves) and right (red curves) elliptically polarized radiation. Panels (a) and (b) show the O K-absorption spectra for the averaged intensity for right and left elliptically polarized radiation. Panels (c) and (d) show the pre-edge region of O K-absorption spectra. Panels (a,c) and (b,d) show results for two different azimuth positions of the sample φ = 0° and φ = 90°, respectively. The spectra were obtained by monitoring the total electron yield by measuring the drain current from the sample. All the spectra were normalized to the continuum jump after subtraction of linear background extrapolated from the energy region below the O1s absorption onset.

Figure 6 shows the O K-absorption spectra measured at three different grazing incidence angles using left and right elliptically polarized radiation and two different azimuth positions (φ = 0° and φ = 90°) of the sample. Figure 6a,b show the O K-absorption spectra for averaged intensity I = 1/2(I_+_ + I_−_) of the right and the left elliptically polarized radiation. Visible anticorrelation between the intensities of the features ***β*** and ***a***-***a*** **′** further supports identification of the feature ***β*** as one related to oxygen deficiency. Surprisingly, the intensity of the feature ***β*** depends strongly on the grazing incidence angle for a certain azimuth position of the sample (panels a and b). Analysis of dependence of the intensity of the features ***β*** on the type of elliptical polarization of the light at fixed incidence angle (panel c and d) reveals strong polarization dependence. The effect is best seen at the smallest incidence angle of 30° while at 90° the intensity of the peak ***β*** barely depends on the light polarization. This observation confirms the initial inference^8^ that the origin of this anisotropy is related to the preferential orientation of spin states involved in the X-ray absorption in the plane parallel to the oxide surface.

### Ti L_2,3_-, NK- and OK-absorption spectra of the TiN electrode

**Figure 7:**
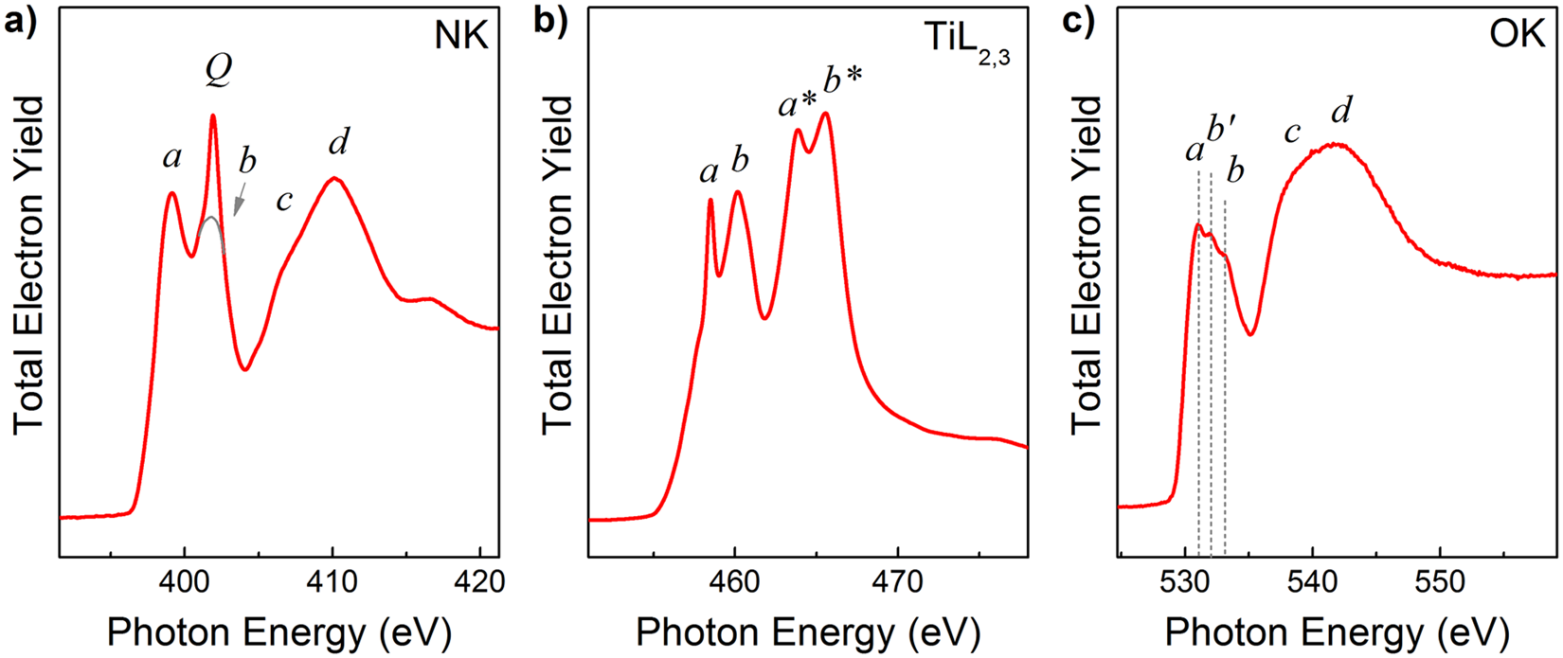
Absorption spectra of the TiN/γ-Al_2_O_3_/Si stack measured through the TiN electrode in the vicinity of (**a**) K- absorption edge of nitrogen; (**b**) L_2,3_- absorption edge of titanium; (**c**) K- absorption edge of oxygen. The grey curve at panel (**﻿a**﻿) shows the model NK-absorption spectrum without contribution of molecular nitrogen peak (i.e., without peak ***Q***). The spectra were obtained by monitoring the total electron yield by measuring drain current from the sample at the incident angle of 45° using s-polarized synchrotron radiation. All the spectra were normalized to the continuum jump after subtraction of linear background extrapolated from the energy region below the corresponding absorption onset.

The absorption spectra of TiN/Al_2_O_3_/Si sample in the vicinity of L_2,3_- absorption edge of titanium and K- absorption edges of nitrogen and oxygen were measured through the TiN electrode are shown in Fig. 7. Since TiN crystallizes in the cubic (rock salt) structure with N atoms occupying interstitial positions in close packed arrangement of Ti atoms, each N atom in TiN is six-fold coordinated. According to the DOS calculations using the localized spherical wave (LSW) method with an extended basis set, the N K -absorption spectrum of TiN consists from the doublet structure (features *a*-*b*) at threshold and the broad structure *c-d* above. The first region *a*-*b* is attributed to the unoccupied N2p states, which are mixed with Ti3d bands. These bands are split by crystal field into the t_2g_ and e_g_ subbands^52, 53^. The wide region *c-d* is attributed to unoccupied N2p states, which are mixed with Ti4sp bands. This region, in particular the shoulder *c*, is sensitive to long range order^54^.

Analysis of the measured N K-spectrum shown in Fig. 7a indicates the presence of intense narrow peak *Q* (around 401.5 eV) in the region of the e_g_ subband. Similarly narrow peak has been found in the earlier works^35, 54–56^. It was shown that during thermal oxidation of TiN at increasing temperatures, oxygen progressively displaces the nitrogen atoms to form TiN_x_O_y_ and, as a result, the peak *Q* was attributed to unbonded nitrogen dissolved in the Ti oxynitride matrix. This conclusion is supported by photoelectron spectroscopy studies discussed above, which confirm formation of unbonded nitrogen in TiN_x_O_y_ layers. From this combined photoelectron spectroscopy and NEXAFS studies we can conclude that formation of unbonded nitrogen is promoted by: i) the oxygen scavenging from the γ-Al_2_O_3_ film by chemically active metal Ti; and 2) oxidation of TiN in air.

Figure 7b shows the Ti L_2,3_-absorption spectrum of the TiN/Al_2_O_3_/Si sample. According to the classical conception, the NEXAFS excitation at the Ti 2p threshold in TiN should reflect the energies of the unoccupied Ti 3d states because it is dominated by the 2p → 3d dipole transitions in the Ti atoms^57^. The measured Ti L_2,3_ absorption spectrum clearly reflects the spin-orbit splitting of the initial Ti 2p level. The Ti 2p_1/2_ structures are marked by asterisks in Fig. 7b. It is well known that the L_2_ absorption spectra is misrepresented by an additional damping channel caused by the L_2_L_3_V- Coster-Kronig transition^58^, which leads to a shorter life time of the L_2_-holes. In this regard, only the L_3_-absorption spectrum will further be discussed.

The features *a* and *b* in Ti L_3_ spectrum of TiN stem from the allowed dipole transitions of Ti 2p_3/2_ electrons to unoccupied 3d states split into 3dt_2g_ (peak *a*) and 3 de_g_ (peak *b*) components by the octahedral surroundings. According to ref. 59, the ligand field splitting effect is weakly expressed (almost absent) in materials with metallic character such as, for example, metallic Ti or c-TiN. The energy separation between features *a* and *b*, which is related to the ligand field splitting, is equal to 1.7 eV (Fig. 7b) which is close to the splitting occurring for TiO_2_ (2.6 eV)^60^. This fact allows one to conclude that some nitrogen atoms in the octahedral surrounding of the Ti atom have been replaced by oxygen atoms.

The O K-absorption spectrum measured on the TiN/Al_2_O_3_/Si sample shown in Fig. 7c provides the additional proof of the TiN electrode oxidation. The O K-absorption spectrum is seen to consist of structured band *a*-*b* and a broad band *c*-*d*. Analysis of the shape and the energy position of main features of the spectrum allows one to notice its similarity with the corresponding spectrum of TiO_2_. Keeping in mind that the O K-absorption spectrum of TiO_2_ originates mainly from O 1s-to-valence transitions, the covalent bonding of the Ti 3d with the O 2p states gives rise to the unoccupied valence t_2g_ and e_g_ orbitals in the octahedral field. Then it is plausible to conclude that the features *a* and *b* in the measured O K-absorption spectrum of the TiN are related to the Ti 3d states mixed with O 2p states^50^. In its turn, the second wide band *c-d* can be attributed to the O2p states mixed with Ti 4sp bands^60^. Note that the first band in the O K-absorption spectrum exhibits a complex structure with three clearly visible features *a*, *b*′, *b*.

**Figure 8:**
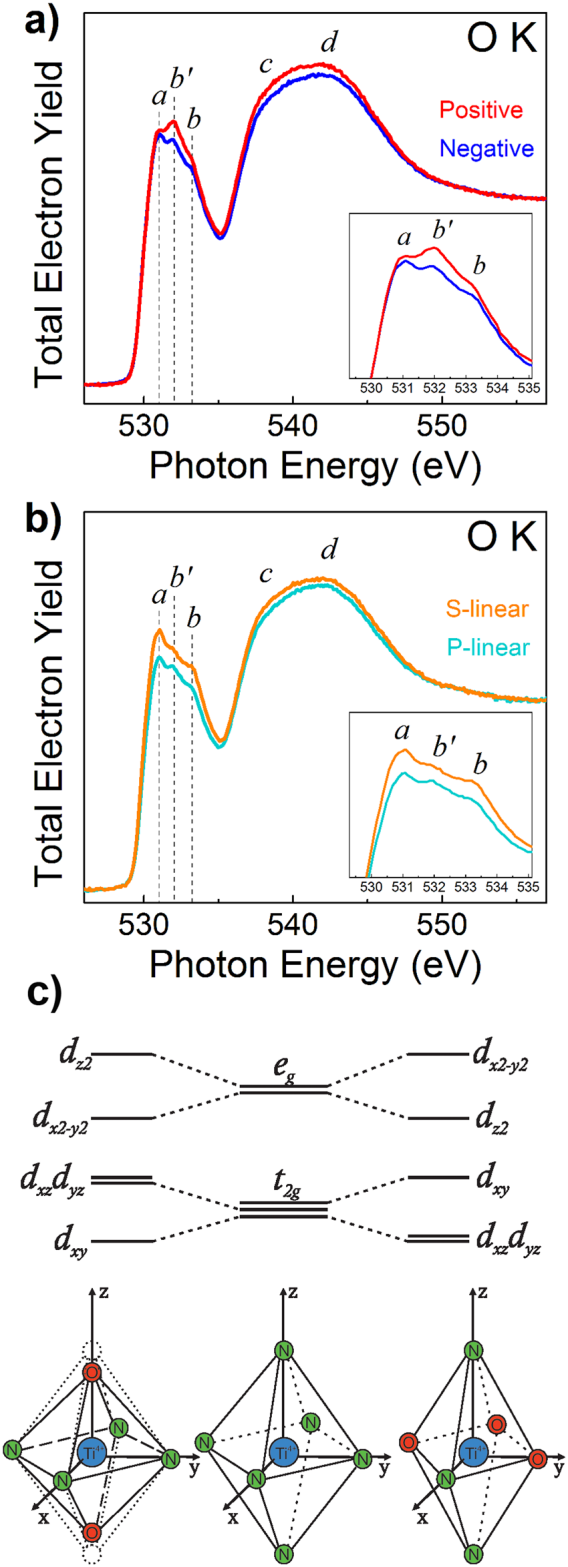
O K-absorption spectra for TiN/Al_2_O_3_/Si stack measured at the grazing incidence angle of 90° using the elliptical (**a**) and linear (**b**) light polarization. All the spectra were normalized to the continuum jump after subtraction of linear background extrapolated from the energy region below the O1s absorption onset. Panel (c) shows schematics of d-state splitting in a perturbed crystal field (compressed and stretched octahedra).

As already mentioned in the introduction, formation of dipole layers can be expected to occur at the interface between dielectric and metal layer due to the charge transfer. To shed some light on the possible mechanism of this effect, the OK-absorption spectra were studied using differently polarized incident light beam. Figure 8 shows the spectra measured at the grazing incidence angle of 90° using the elliptical (a) and linear (b) light polarization. One can see that the OK-absorption spectra show the largest polarization sensitivity in the case of positive and negative elliptical polarizations (the intensity of the feature *b*′ varies depending on the polarization).

Since in octahedral complexes the metal e_g_ orbitals are directed towards the corners (ligand anions) of the octahedra, they should have a stronger overlap with orbitals of the neighboring atoms (2p orbitals of oxygen atoms). As a result, the double degenerate e_g_ state (d_z_^2^ and d_x_^2^_−y_^2^ components) appears to be more sensitive (as compared to t_2g_ states) to deviations from the octahedral symmetry^60–63^. The replacement of nitrogen atoms by oxygen ones in the nearest surroundings of the Ti atom unavoidably affects the short range Ti environment leading to tetragonal distortion of the original octahedral symmetry and to splitting of both t_2g_ and e_g_ states. It is important that the deformation of the octahedron is possible both along Z axis and in the XY plane. Would the oxygen atom substitute the nitrogen atom at the vertices of the octahedron along Z axis direction, compression of the octahedron will occur making d_xy_ and d_x_^2^_−y_^2^ orbitals more sensitive to the changes in the nearest surroundings of the titanium atom. These orbitals will then be energetically located below d_xz_, d_yz_ and d_z_^2^ states. In the case of octahedron deformation in the XY plane, the decrease of the Ti-O distance in the XY plane relative to the Ti-N distance leads to the octahedron “stretched” along the Z axis and resulting in the opposite direction of energy shift of the orbitals inside t_2g_ and e_g_ states. As a result, the d_xz_, d_yz_ and d_z_^2^ orbitals will become more sensitive to the changes in the nearest surrounding of the titanium atom and making these states more energetically favorable as compared to the “stretched” octahedron. This distortion of TiN_6−n_O_n_ octahedra leading to the mixing of (d_z_^2^, d_x_^2^_−y_^2^) and (d_x_^2^_−y_^2^, d_z_^2^) states is reflected in the experimentally observed behavior of the peaks *b*′-*b* in the spectra shown in Figs 7c and 8a,b.

The O K-absorption spectra measured using elliptically polarized radiation are compared in Fig. 8a. One can see that in positive elliptical radiation geometry the intensity of the low energy component (*b*′) of e_g_ state is more pronounced. Since the d_z_^2^ orbital has stronger directionality in space than d_x_^2^_−y_^2^ one, the intensity of transition to the d_z_^2^ state should be the most sensitive to the light polarization change. Therefore, the most polarization-sensitive *b*′ feature should be attributed to the contribution of the d_z_^2^ state to OK absorption spectrum. Because the *b*′ feature has lower energy position than the *b* feature, the established trend allows us to propose that predominantly “stretched” TiN_6−n_O_n_ octahedra with the preferential orientation in the space are formed at the TiN/γ-Al_2_O_3_ interface due to oxygen re-distribution. Worth of adding here is that Ti L_2,3_-absorption spectra (not shown) exhibit no measurable sensitivity to polarization of the light.

Next, if we address the O K-absorption spectra measured using linear light polarization (Fig. 8b), the dependence of the intensity of components of t_2g_ and e_g_ states on the s- and p- type of polarization can also be traced but it appears to remain weak. One can see that while the total intensity of the *a*-*b* band changes, the intensity of the *b*′ feature becomes more pronounced in the case of p-polarized light. This kind of anisotropy is also consistent with preferential orientation of the oxidation-induced TiN lattice distortion. Let us now examine the impact of this distortion on the electrostatic potential distribution across the TiN/γ-Al_2_O_3_ interface by observing photoemission of electrons from the states near the Fermi level of TiN in the oxide conduction band.

### Internal photoemission analysis of TiN/γ-Al_2_O_3_ interface barriers

**Figure 9:**
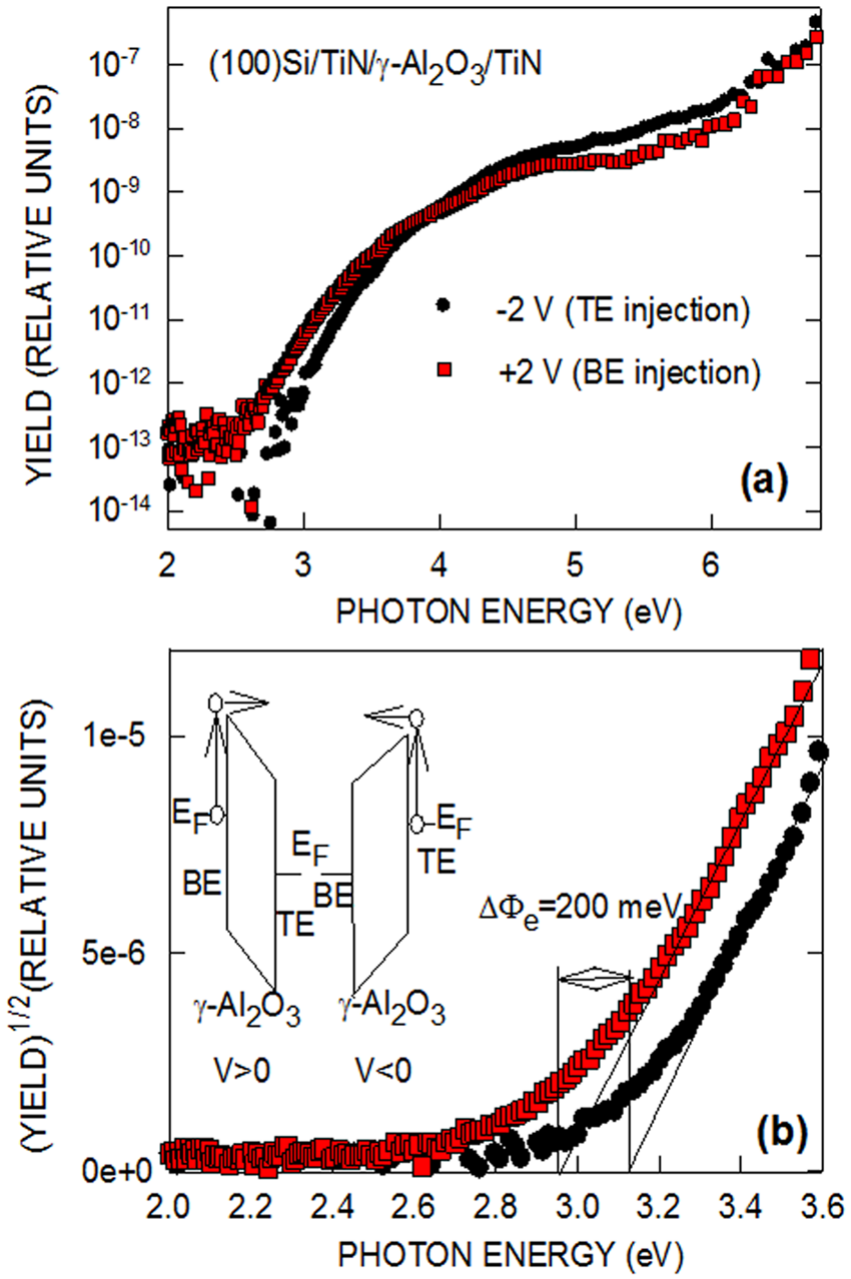
Semi-logarithmic (top) and Fowler (bottom) plots as measured on the TiN/γ-Al_2_O_3_/TiN sample under the indicated bias voltage applied to the top TiN electrode. The observed signal corresponds to electron IPE from the bottom electrode (BE) and top electrode (TE), respectively, as illustrated in the inset in bottom panel.

The IPE yield spectra as measured on the TiN/γ-Al_2_O_3_/TiN sample are shown in Fig. 9 as semi-logarithmic (a) and Fowler (b) plots. The signal observed under positive and negative bias voltage V applied to the top TiN electrode corresponds to electron IPE from the bottom electrode (BE) and top electrode (TE), respectively. The corresponding electron transitions from TiN into the CB of γ-Al_2_O_3_ are schematically illustrated in the inset in panel (b). There is clearly visible difference in the observed IPE spectral threshold of ≈200 meV while no significant difference in the quantum yield can be seen far (1–2 eV) above the threshold. This observation suggests that the difference in the energy barrier, which electrons encounter at the top and bottom TiN/γ-Al_2_O_3_ interfaces, is unlikely to be caused by charges located in the oxide layer since the latter would cause additional electron scattering and the corresponding reduction of the electron photoemission yield. Rather, this behavior points towards EWF difference between TiN TE and BE related to different composition of the near-interface metal and oxide layers. This explanation is in agreement with the above described results indicating formation of a 1.1-nm thick oxidized TiN IL at the top TiN/γ-Al_2_O_3_ interface.

Worth of noticing here is that deviation of Fowler plots from the ideal (linear) behavior [cf. panel (b)] suggests significant spread of the barrier values. The latter would be consistent with the formation of laterally nonuniform interface barrier due local oxidation of TiN, e.g., enhanced at the grain boundaries. Nevertheless this barrier nonuniformity is marginally affected by the O-deficiency development at the interface as no substantial difference in the “tail” region is observed between the top and bottom TiN/γ-Al_2_O_3_ interfaces. At the same time, the observed interface barrier increase by ≈200 meV at the O-deficient top TiN/γ-Al_2_O_3_ interface as compared to the bottom one (formed during ALD under abundant oxidant supply) indicated in Fig. 9 makes the effects related to formation of amorphous alumina unlikely: Would amorphization of Al_2_O_3_ occur, the TiN/Al_2_O_3_ interface barrier should become ≈500 meV lower^18^. Therefore, we ascribe the observed barrier variation to the charge transfer caused by distortion of crystalline units of the contacting materials as revealed by HAXPES and NEXAFS experiments. As the major conclusion, these results indicate that “symmetric” TiN/γ-Al_2_O_3_/TiN structure appears to be asymmetric in terms of interface barrier height leading to a non-zero built-in potential. We hypothesize that formation of anisotropic structures at both sides of the TiN/γ-Al_2_O_3_ interface leads to the interface charge transfer eventually resulting in the metal effective work function changes.

### Conclusions

The presented results indicate that the TiN electrodes in TiN/γ-Al_2_O_3_/TiN stacks are multilayered systems. It is important that the formation of TiN_x_O_y_ layers occurred from two sides of the top TiN electrode but due to different processes: oxygen scavenging from the γ-Al_2_O_3_ film and oxidation from ambient. The implementation of HAXPES method allows us to estimate the thickness of all the layers constituted TiN electrode, which can be correlated with different height of electron barrier at the top and bottom interfaces of the TiN/γ-Al_2_O_3_/TiN stacks. The most important is that the formation of polarized layer near the surface in the exposed γ-Al_2_O_3_ was observed and we assert that the exact origin of this anisotropy is related to the preferential orientation of spin states involved in the X-ray absorption in the plane of the surface. The investigation of the electrode reveals the existence of spatial and angular dichroism in the structure of TiN manifested in the formation of predominantly “stretched” octahedra with the preferential orientation relative the interface in the studied sample.

## Methods

**Figure 10:**
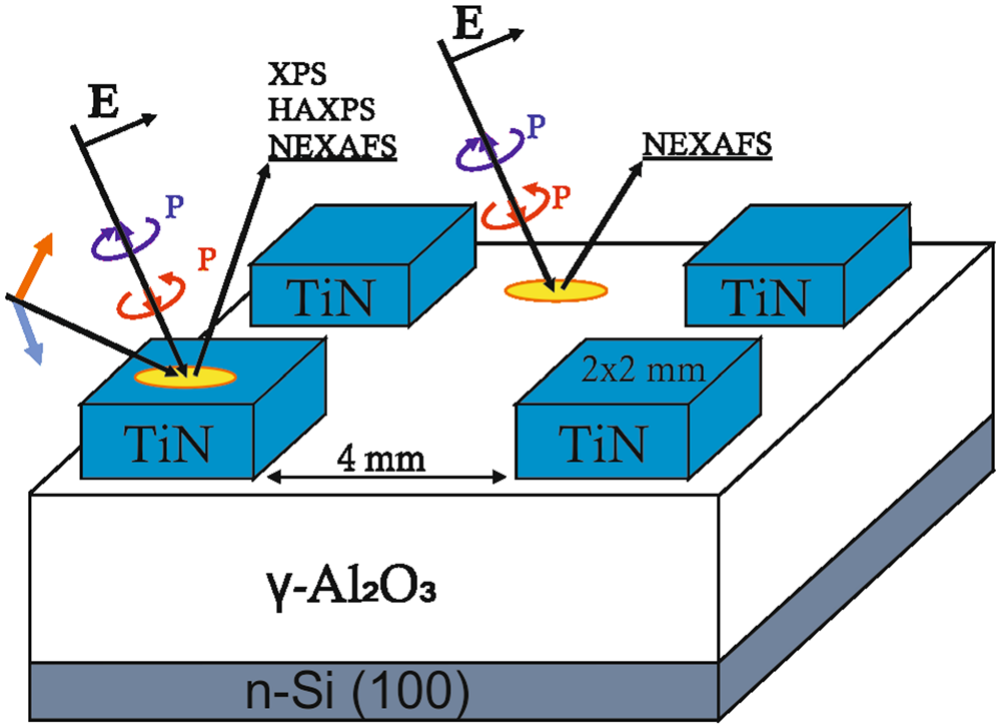
Schematic representation of TiN/γ-Al_2_O_3_/Si sample after patterning TiN electrodes illustrating geometry of applying different measurement techniques.

The studied TiN/γ-Al_2_O_3_/TiN/Si, TiN/γ-Al_2_O_3_/Si structures were fabricated on 300 mm (100)Si wafers using oxide atomic layer deposition (ALD) from Al(CH_3_)_3_ and H_2_O precursors at 300 °C followed by physical vapor deposition of TiN on unheated substrate. Thicknesses of the oxide and the metal layers were 17 (or 12) and 10 nm, respectively. Prior to the top metal deposition, the Al_2_O_3_ layers grown on the bottom TiN electrode were crystallized to cubic (γ-) phase by 1-min 1000 °C anneal in N_2_, the process relevant to flash cell processing^16^. Alternatively, sample fabricated on bare Si substrates were annealed in O_2_ atmosphere at 1100 °С for 60 seconds to ensure crystallization in γ-phase without oxygen loss. Next, the TiN layer of 10 nm thickness was deposited on top of the crystallized oxide. Importantly, prior to each metallization step the samples were *in situ* degassed by applying 5 min 380 °C anneal in high vacuum to ensure removal of adsorbates from the sample surface. Therefore, the oxygen balance at the studied interfaces is determined by its supply from the oxide film. Finally, the top TiN layer was patterned using HBr/Cl plasma to form large (2 mm^2^) MOS and MIM capacitors enabling both electrical measurements and physical characterization (Fig. 10). No damage of γ-Al_2_O_3_ was detected after plasma patterning, details can be found in ref. 8.

Electrical characterization was aiming at determination of energy barrier for electrons at TiN/γ-Al_2_O_3_interfaces using internal photoemission (IPE) of electrons from the states near the Fermi level of the metal into the oxide CB^64^. The IPE spectra were measured in the photon energy (hν) range from 2 to 6.6 eV. The quantum yield of IPE (Y) was defined as the photocurrent normalized to the incident photon flux. From the experimental Y-hν spectral curves the height of the interface barrier was obtained using linear fitting of the Fowler, i.e., Y^1/2^-hν plot^65^.

Structural and chemical characterization was done using X-ray photoelectron spectroscopy (XPS), X-ray photoelectron spectroscopy with high kinetic energies (HAXPES), and the near edge X-ray absorption fine structure (NEXAFS). The implementation of HAXPES has allowed us to carry out in-depth analysis of chemical composition and to reconstruct the thicknesses of all the layers constituting the TiN electrodes.

TiN/γ-Al_2_O_3_/Si sample was analyzed in both the area of uncovered γ-Al_2_O_3_ film exposed between the electrodes and through the top TiN electrode. The distance between the electrodes was ∼4 mm, which exceeds the horizontal size of the light spot in the NEXAFS analysis. The spot size was about 0.6 × 0.8 mm (vertical × horizontal) that also allowed us to study the electrode itself and the interface through the electrode. The probing depth in the total electron yield method is approximately 10 nm^66, 67^ that allowed us to reliably detect signals from the TiN/γ-Al_2_O_3_ interface. The TiN/γ-Al_2_O_3_/Si sample was studied additionally through the electrode using XPS and HAXPES methods. The absence of the contribution stemming from the γ-Al_2_O_3_ film outside the electrode during the study through the electrode was controlled by the absence of Al 2 s–photoelectron line in the HAXPES spectrum measured at excitation energy of 2010 eV when the escape depth of the photoelectrons is smaller than the thickness of the TiN electrode at any electron emission angle.

NEXAFS, XPS, and HAXPES measurements were performed at the BESSY II synchrotron light source at the Helmholtz-Zentrum Berlin. The NEXAFS measurements were carried out using differently polarized synchrotron radiation at the Polarimeter station at UE56/2 PGM-2 beamline by monitoring the total electron yield from the samples in a current mode. The GaAsP diodes were used as X-ray detectors with a Keithley 617 electrometer as a current meter. The absolute energy calibration was carried out by measuring the energies of the reference N_2_ lines as well as of the absorption edges for beryllium, titanium and iron radiation filters in the first and higher orders of diffraction. The attained energy resolution was better than E/ΔE = 3000 with the absolute accuracy of the energy scale of about 0.1 eV. The relative intensities of NEXAFS Al L_2,3_- and O K- absorption spectra have been normalized to the continuum jump at the photon energy of 120 eV and 566 eV, respectively, after subtraction of a background linearly extrapolated from the energy region below the absorption onset. This normalization procedure provides about the same total oscillator strength for Al L_2,3_- and O K-absorption spectra over the photon energy range of 76–88 eV and 525–566 eV, respectively, which is consistent with a general behavior of oscillator strength distribution for the atomic X-ray absorption^57^.

The XPS measurements were performed at RGL-station on the Russian-German beamline. The XPS spectra were taken at the excitation photon energy of 700 eV using a hemispherical electron energy analyzer (Specs Phoibos 1500). All the photoemission spectra were collected with the combined analyzer and monochromator energy resolution better than 430 meV.

The HAXPES experiments were performed using the HIKE station at the KMC-1 beamline. This experimental setup is equipped with a high-resolution hemispherical electron analyzer optimized for high-energy electrons (VG Scienta R4000). The analyzer is installed at 90° to the incident beam, i.e., the electron emission is detected in the direction along the normal to the sample surface and grazing incidence of the photon beam. The analyzer resolution was set to 0.25 eV while the KMC-1 double-crystal monochromator provided a 0.4 eV bandwidth at 3010 eV. Further details about the experimental setup can be found in refs 68 and 69. The binding energy scale both in XPS and HAXPES experiments was referenced to the Fermi energy of TiN by combined measurements of the core levels and the valence band spectra and setting the Fermi energy to zero.

## References

[1] Kornblum L (2012). Investigation of the band offsets caused by thin Al_2_O_3_ layers in HfO_2_ based Si metal oxide semiconductor devices. Appl. Phys. Lett..

[2] Guha S, Narayanan V (2007). Oxygen vacancies in high dielectric constant oxide-semiconductor films. Phys. Rev. Lett..

[3] Jagadeesh Chandra SV, Fortunatoa E, Martinsa R, Choi C-J (2012). Modulations in effective work function of platinum gate electrode in metal-oxide-semiconductor devices. Thin Solid Films.

[4] Schaeffer JK (2004). Contributions to the effective work function of platinum on hafnium dioxide. Appl. Phys. Lett..

[5] Kolomiiets NM, Afanas’ev VV, Opsomer K, Houssa M, Stesmans A (2016). Hydrogen induced dipole at the Pt/oxide interface in MOS devices. Phys. Stat. Sol..

[6] Afanas’ev VV (2011). TiN_x_/HfO_2_ interface dipole induced by oxygen scavenging. Appl. Phys. Lett..

[7] Pantisano L (2011). Towards barrier height modulation in HfO_2_/TiN by oxygen scavenging – Dielectric defects or metal induced gap states?. Microelectron. Eng..

[8] Filatova EO, Konashuk AS, Schaefers F, Afanas’ev VV (2016). Metallization-induced oxygen deficiency of γ-Al_2_O_3_ layers. J. Phys. Chem. C.

[9] Pantisano L (2006). Effective work function modulation by controlled dielectric monolayer deposition. Appl. Phys. Lett..

[10] D Stefano Fr (2014). Modulation of electron barriers between TiN_x_ and oxide insulators (SiO_2_, Al_2_O_3_) using Ti interlayer. Phys. Status Solidi A.

[11] Monch, W. Semiconductor Surfaces and Interfaces. (Springer, 1993).

[12] Wang CG, DiStefano TH (1975). Polarization layer at metal/insulator interfaces. CRC Critical Rev. Sol. State Sci..

[13] Kurtin S, McGill TC, Mead CA (1969). Fundamental transition in the electronic nature of solids. Phys. Rev. Lett..

[14] Mead CA, Snow EH, Deal BE (1966). Barrier lowering and field penetration at metal/dielectric interfaces. Appl. Phys. Lett..

[15] Kittl JA (2009). High-k dielectrics for future generation memory devices. Microelectronic Eng..

[16] Zahid MB (2010). Applying complementary trap characterization technique to crystalline γ-phase-Al_2_O_3_ for improved understanding of nonvolatile memory operation and reliability. IEEE Trans. Electron. Dev..

[17] Afanas’ev VV, Stesmans A, Mrstick BJ, Zhao C (2002). Impact of annealing-induced densification on electronic properties of atomic-layer-deposited Al_2_O_3_. Appl. Phys. Lett..

[18] Afanas’ev VV (2011). Influence of Al_2_O_3_ crystallization on band offsets at interfaces with Si and TiN_x_. Appl. Phys. Lett..

[19] Toyoda S, Shinohara T, Kumigashira H, Oshima M, Kato Y (2012). Significant increase in conduction band discontinuity due to solid phase epitaxy of Al_2_O_3_ gate insulator films on GaN semiconductor. Appl. Phys. Lett..

[20] Tanner CM (2007). Engineering epitaxial gamma-Al_2_O_3_ gate dielectric films on 4H-SiC. J. Appl. Phys..

[21] Correa SA (2009). Enhancement in interface robustness regarding thermal oxidation in nanostructured Al_2_O_3_ deposited on 4H-SiC. Appl. Phys. Lett..

[22] Jakschik S (2003). Crystallization behavior of thin ALD-Al_2_O_3_ films. Thin Solid Films.

[23] Park JK (2011). Mechanism of date retention improvement by high temperature annealing of Al_2_O_3_ blocking layer in flash memory device. Jpn. J. Appl. Phys..

[24] Specht (2005). Charge trapping memory structures with Al_2_O_3_ trapping dielectric for high-temperature applications. Solid State Electron..

[25] Xu ZG, Zhu C, Huo Z, Zhao S, Liu M (2012). Effects of high-temperature O_2_ annealing on Al_2_O_3_ blocking layer and Al_2_O_3_/Si_3_N_4_ interface for MANOS structures. J. Phys. D: Appl. Phys..

[26] Powell CJ, Jablonski A, Tilinin IS, Tanuma S, Penn DR (1999). Surface sensitivity of auger-electron spectroscopy and X-ray photoelectron spectroscopy. J. Electron Spectrosc. Relat. Phenom..

[27] Tanuma S, Powell CJ, Penn DR (1994). Calculations of electron inelastic mean free paths. V. data for 14 organic compounds over the 50–2000 eV range. Surf. Interface Anal..

[28] HIKE technical details. *Helmholtz Zentrum Berlin site* https://www.helmholtz-berlin.de/pubbin/igama_output?modus=datei&did=147 (2012).

[29] Milosev I, Strehblow H-H, Navinsek B, Metikos-Hukovic M (1995). Electrochemical and thermal oxidation of TiN coatings studied by XPS. Surf. Interface Anal..

[30] Glaser A (2007). Oxidation of vanadium nitride and titanium nitride coatings. Surf. Sci..

[31] Saha NC, Tompkins HG (1992). Titanium nitride oxidation chemistry: an X-ray photoelectron spectroscopy study. J. Appl. Phys..

[32] Bertoti I, Mohai M, Sullivan JL, Saied SO (1995). Surface characterisation of plasma-nitrided titanium: an XPS study. Appl. Surf. Sci..

[33] Bruninx E, Van Eenbergen AFPM, Van Der Werf P, Haisma J (1986). X-ray photoelectron spectroscopy of hafnium nitride. J. Mat. Sci..

[34] Pulsipher DJV, Martin IT, Fisher ER (2010). Controlled nitrogen doping and film colorimetrics in porous TiO_2_ materials using plasma processing. ACS Appl. Mater. Interfaces.

[35] Esaka F (1997). Comparison of surface oxidation of titanium nitride and chromium nitride films studied by X-ray absorption and photoelectron spectroscopy. J. Vac. Sci. Technol. A.

[36] Tougaard S (1997). Universality classes of inelastic electron scattering cross-sections. Surf. Interface Anal..

[37] Jaeger D, Patscheider JA (2012). Complete and self-consistent evaluation of XPS spectra of TiN. J. Electron Spectrosc. Relat. Phenom..

[38] Porte L, Roux L, Hanus J (1983). Vacancy effects in the X-ray photoelectron spectra of TiN_x_. Phys. Rev. B..

[39] Wallbank B, Main IG, Johnson CE (1974). 2p and 2s shake-up satellites in solid compounds of 3d ions. J. Electron Spectrosc. Relat. Phenom..

[40] Filatova EO (2012). Soft X-ray reflectometry, hard X-ray photoelectron spectroscopy and transmission electron microscopy investigations of the internal structure of TiO_2_(Ti)/SiO_2_/Si stacks. Sci. Technol. Adv. Mater..

[41] Moulder, J. F. *Handbook of X-ray Photoelectron Spectroscopy* (Perkin-Elmer Corp., Physical Electronics Division, 1992).

[42] Filatova, E. O., Kozhevnikov, I. V. & Sokolov, A. A. Characterization of High-k Dielectrics Internal Structure by X-ray Spectroscopy and Reflectometry New Approaches to Inter Layer Identification and Analysis, In: Gang, H., Zhaoqi, S. High-k Gate Dielectrics for CMOS Technology (Wiley-VCH Verlag, 2012).

[43] Trzhaskovskaya MB, Nefedov VI, Yarzhemsky VG (2001). Photoelectron angular distribution parameters for elements Z = 1 to Z = 54 in the photoelectron energy range 100–5000 eV. At. Data Nucl. Data Tables.

[44] Britov IA, Romashenko YN (1978). X-ray spectroscopic investigation of electronic structure of silicon and aluminum oxides. Phys. Solid State.

[45] Konashuk AS, Sokolov AA, Drozd VE, Schaefers F, Filatova EO (2013). Study of Al_2_O_3_ nanolayers synthesized onto porous SiO_2_ using X-ray reflection spectroscopy. Thin Solid Films.

[46] Filatova EO, Konashuk AS (2015). Interpretation of the changing the band gap of Al_2_O_3_ depending on its crystalline form: connection with different local symmetries. J. Phys. Chem. C.

[47] Konashuk AS, Sokolov AA, Drozd VE, Romanov A, Filatova EO (2012). The influence of porous silica substrate on the properties of alumina films studied by X-ray reflection spectroscopy. Techn. Phys. Lett..

[48] Bokhoven JA, Nabi T, Sambe H, Ramaker DE, Koningsberger DC (2001). Interpretation of the Al K- and L_II/III_-edges of aluminium oxides: differences between tetrahedral and octahedral Al explained by different local symmetries. J. Phys.: Condens. Matter.

[49] Ching WY, Ouyang L, Rulis P, Yao H (2008). Ab initio study of the physical properties of γ-Al_2_O_3_: lattice dynamics, bulk properties, electronic structure, bonding, optical properties, and ELNES/XANES spectra. Phys. Rev. B: Condens. Matter Mater. Phys..

[50] Filatova EO (2013). X-ray spectroscopic study of SrTiO_x_ films with different interlayers. J. Appl. Phys..

[51] Muller DA, Nakagawa N, Ohtomo A, Grazul JL, Hwang HY (2004). Atomic-scale imaging of nanoengineered oxygen vacancy profiles in SrTiO_3_. Nature.

[52] Ballhausen, C. J. *Introduction to Ligand Field Theory* (McGraw-Hill, 1962).

[53] Figis, B. N. *Introduction to Ligand Fields* (Wiley, 1966).

[54] Soriano L, Abbate M, Pen H, Czyzyk MT, Fuggle JC (1993). The interaction of N with Ti and the oxidation of TiN studied by soft X-ray absorption spectroscopy. J. Electron Spectrosc. Relat. Phenom..

[55] Soriano L (1993). Thermal oxidation of TiN studied by means of soft X-ray absorption spectroscopy. J. Vac. Sci. Technol. A.

[56] Klimczyk P (2004). Cubic boron nitride—Ti/TiN composites: hardness and phase equilibrium as function of temperature. J. Alloys Compd..

[57] Fano U, Cooper JW (1968). Spectral distribution of atomic oscillator strength. Rev. Mod. Phys..

[58] Grebennikov VI, Galakhov VR, Finkel’shtein LD, Ovechkina NA, Kurmaev EZ (2003). Effect of atomic magnetic moments on the relative intensity of the L_β_ and L_α_ components in X-ray emission spectra of 3d transition metal oxides. Phys. Solid State.

[59] Fink J (1985). 2p absorption spectra of the 3d elements. Phys. Rev. B..

[60] de Groot FMF, Fuggle JC, Thole BT, Sawatzky GA (1990). L_2,3_ X-ray-absorption edges of d^0^ compounds: K^+^, Ca^2+^, Sc^3+^, and Ti^4+^ in O_h_ (octahedral) symmetry. Phys. Rev. B.

[61] Mastelaro VR (2006). Electronic structure of Pb_1−x_La_x_TiO_3_ ferroelectric materials from Ti 2p and O 1s soft X-ray absorption spectroscopy. J. Appl. Phys..

[62] Sugano, S., Tanabe, Y. & Kamimura, H. *Multiplets of transition-metal ions in crystals* (Academic press, 1970).

[63] Bersuker, I. B. Electronic Structure and Properties of Coordination Compounds (Khimiya, 1976).

[64] Afanas’ev VV, Stesmans A (2007). Internal photoemission at interfaces of high-κ insulators with semiconductors and metals. J. Appl. Phys..

[65] Powell RJ (1971). Interface barrier energy determination from voltage dependence of photoinjected currents. J. Appl. Phys..

[66] Stöhr, J. *NEXAFS Spectroscopy* (Springer, 1992).

[67] Hähner G (2006). Near edge X-ray absorption fine structure spectroscopy as a tool to probe electronic and structural properties of thin organic films and liquids. Chem. Soc. Rev..

[68] Gorgoi M (2009). The high kinetic energy photoelectron spectroscopy facility at BESSY progress and first results. Nucl. Instrum. Methods Phys. Res. A.

[69] Schaefers F, Mertin M, Gorgoi M (2007). KMC-1: A high resolution and high flux soft X-ray beamline at BESSY. Rev. Sci. Instrum..

